# “From me to HIV”: a case study of the community experience of donor transition of health programs

**DOI:** 10.1186/s12879-015-1068-8

**Published:** 2015-08-19

**Authors:** Daniela C. Rodríguez, Vandana Tripathi, Meghan Bohren, Amy Paul, Kriti Singh, Vibha Chhabra, Suneeta Singh, Sara Bennett

**Affiliations:** Department of International Health, Johns Hopkins Bloomberg School of Public Health, 615 N. Wolfe Street, Rm. E-8612, Baltimore, MD 21205 USA; Department of Population, Family, and Reproductive Health, Johns Hopkins Bloomberg School of Public Health, Baltimore, USA; Department of Health Policy and Management, Johns Hopkins Bloomberg School of Public Health, Baltimore, USA; Amaltas Consulting Pvt Ltd, Delhi, C 20 Hauz Khas, New Delhi, 110 016 India

## Abstract

**Background:**

Avahan, a large-scale HIV prevention program in India, transitioned over 130 intervention sites from donor funding and management to government ownership in three rounds. This paper examines the transition experience from the perspective of the communities targeted by these interventions.

**Methods:**

Fifteen qualitative longitudinal case studies were conducted across all three rounds of transition, including 83 in-depth interviews and 45 focus group discussions. Data collection took place between 2010 and 2013 in four states: Andhra Pradesh, Karnataka, Maharashtra and Tamil Nadu.

**Results:**

We find that communication about transition was difficult at first but improved over time, while issues related to employment of peer educators were challenging throughout the transition. Clinical services were shifted to government providers resulting in mixed experiences depending on the population being targeted. Lastly, the loss of activities aimed at community ownership and mobilization negatively affected the beneficiaries’ view of transition.

**Conclusions:**

While some programmatic changes resulted in improvements, additional opportunity costs for beneficiaries may pose barriers to accessing HIV prevention services. Communicating and engaging community stakeholders early on in future such transitions may mitigate negative feelings and lead to more constructive relationships and dialogue.

## Background

**Fig. 1:**
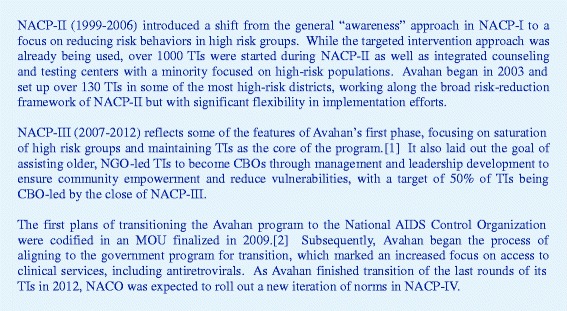
Avahan and the National AIDS Control Program (NACP) [2]

Avahan, a large-scale HIV prevention intervention in India funded by the Bill and Melinda Gates Foundation (BMGF), was established in 2003 with programs in four southern and two northeastern states (Andhra Pradesh, Karnataka, Maharashtra, Manipur, Nagaland and Tamil Nadu). Through local implementing partners, Avahan established over 130 targeted interventions (TIs) focused on serving key populations (KPs), including female sex workers (FSW), men who have sex with men (MSM), transgendered individuals (TGs), clients of sex-workers along trucking routes, and injecting drug users. From the time of inception, BMGF planned to transition Avahan TIs to the Government of India for continued management and funding (see Fig. 1) [3]. The transition took place in three tranches with 10 % of TIs transitioning in 2009, 20 % in 2011 and 70 % in 2012.

**Fig. 2:**
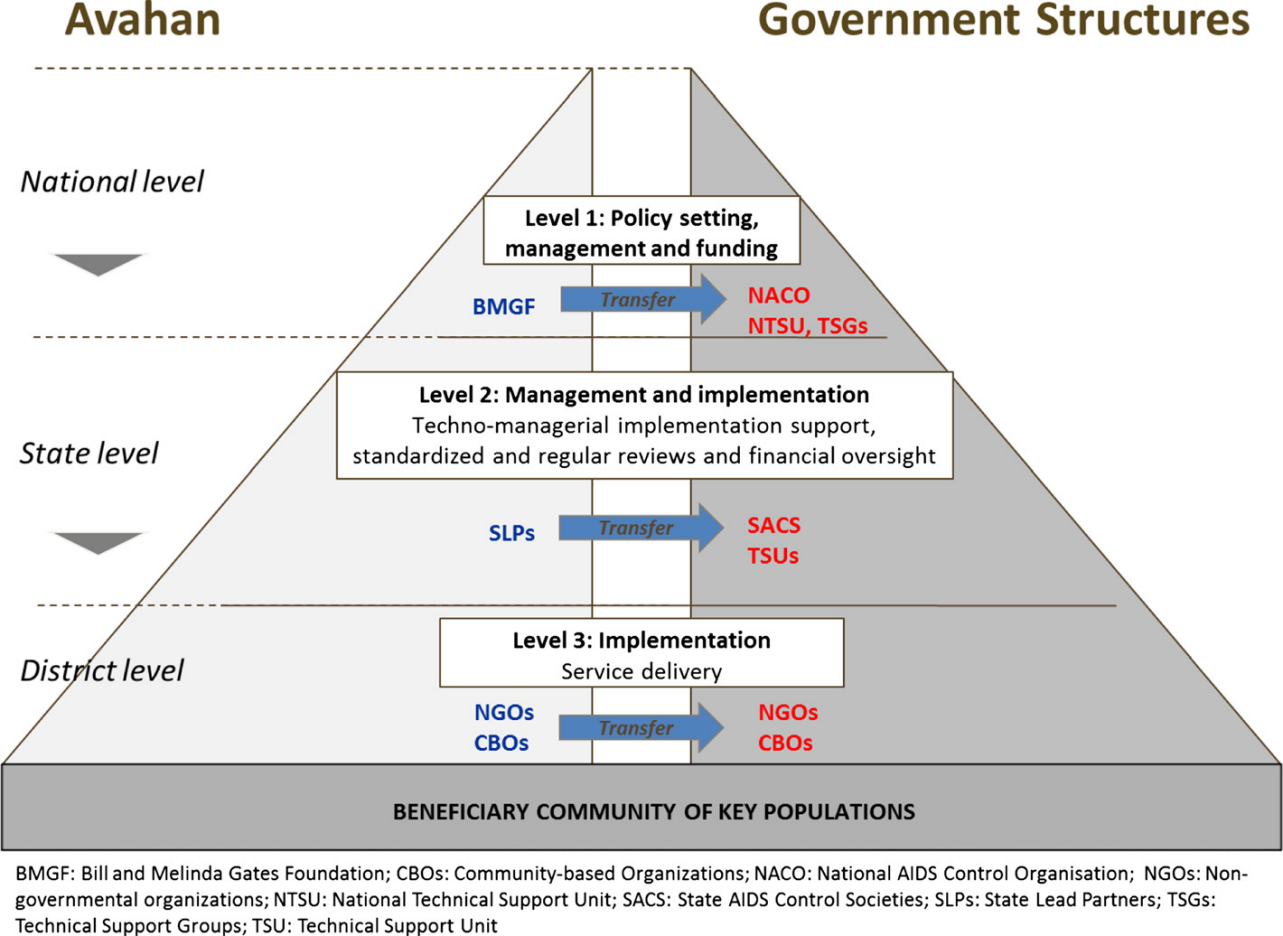
Stakeholders involved in transition of Avahan program to Government of India

The transition of Avahan TIs to government involved the transfer of supervision responsibilities as well as TI management to government structures and funding (see Fig. 2). At the national level, the management responsibilities were transferred from BMGF to the National AIDS Control Organisation (NACO) and national technical support units (NTSUs) aimed at supporting the implementing agencies. At the state level, Avahan was administered by state lead partners (SLPs) which were large non-governmental organizations (NGOs) with experience in local program implementation, including national and international NGOs and academic institutions. As part of transition, the SLPs’ responsibilities were transferred to State AIDS Control Societies (SACS) and Technical Support Units (TSUs) in each state. Small, local NGOs and community-based organizations (CBOs), which were implementing TI programs under Avahan, were typically re-contracted by SACS.

In preparation for transition, Avahan TIs aligned with the National AIDS Control Program (NACP) guidelines, which resulted in changes to certain aspects of program implementation. In terms of clinical services, Avahan TIs were more focused on overall health with a significant component targeting the management of sexually transmitted infections (STIs). Alignment with government guidelines resulted in greater focus on HIV testing. Also, while most Avahan TIs had project-owned clinics or links to private providers for delivery of various medical services, the post-transition model generally directed KPs to government health facilities for testing, care and treatment for HIV only.

Another core component of the Avahan program was services for local communities, including outreach and health education. Avahan invested heavily in activities that could facilitate social change, such as police sensitization and crisis response teams that were available to KPs in case of harassment, police raids, or arrest. Lastly, Avahan emphasized activities that mobilized the community and empowered them to establish local CBOs to manage their own TIs.

The goal of the transition was to ensure that the TIs’ activities continued and their contribution to a sustained HIV response was not lost. A key aspect of this goal is the experience of the beneficiary community to whom these services were targeted. KPs represent the end users of the services being offered and front-line staff, specifically peer educators (PEs), is drawn predominantly from these groups. These front-line workers play a dual role in the TI due to their responsibilities in delivering services, such as outreach and engagement, but also as KPs themselves. The KP experience provides a user’s perspective of the transition process, which is crucial to understanding the likely sustainability of service utilization and, in turn, the sustainability of gains in HIV prevention.

There has been limited previous study of program transitions from the perspective of affected communities. Past evaluations of community initiatives in the U.S. have focused primarily on program continuation [4, 5] and less frequently on the quality of the activities that continue to be implemented [6]. Even where sustainability was evaluated favorably, these experiences showed changes in available funding, challenges retaining staff, and shifting focus due to changing community needs or to better “fit” with new sources of support [4, 5]. In the developing world, several community initiatives have been evaluated for sustainability post donor funding. A community program for disabled people in Ghana showed failure to continue interventions despite local government having allocated funds to do so, largely owing to lack of capacity to use funds and low interest from local government systems [7]. In their experience post-World Bank funding, CBO representatives from Zambia described maintaining function but facing significant challenges in retaining paid staff and shifting emphasis across different health issues [8].

This paper presents the findings from a series of longitudinal case studies of TIs which explored in detail the transition experience from the perspective of the involved stakeholders, including the beneficiary community. The case studies also assessed unanticipated consequences and dynamic changes provoked by the transition. Given the paucity of evidence regarding community experience of program transition, the focus of this paper is to understand how the transition of Avahan financing and management responsibility to government was experienced by the beneficiary community. The paper explores experiences of the transition process itself, as well as changes to the character of the program that took place due to and following transition.

## Methods

These case studies form part of a larger evaluation of the Avahan transition process described elsewhere [9]. They were designed to assess the experience of TIs transitioning in each transition tranche in the Southern states.^1^

A total of 15 TIs were studied over four rounds of data collection. Four TIs that transitioned in 2009 and 2011 were revisited in 2012/2013 to follow up on their experiences after transition. TIs were chosen purposively to provide variation based on key characteristics, such as state, high-risk group (HRG) served, SLP, implementing organization, etc. (see Table 1). One additional case study was completed on a TI working with long-distance truckers. Given the different nature of the TI and its beneficiary community, its explanatory value for this paper is limited and thus was excluded.

**Table 1:** Sample of case study TIs and revisited TIs

TI Characteristics^a^		Andhra Pradesh	Karnataka	Maharashtra	Tamil Nadu	Total
HRG	FSW	1	1	4	-	6
	MSM/TGs	2	1	-	2	5
	Composite^b^	1	2	-	1	4
Implementer	NGO	3	1	4	1	9
	CBO	1	3	-	2	6
Tranche of Transition	2009	1	1	1	1	4
	2011	1	2	1	1	5
	2012	2	1	2	1	6
	Revisits	1 (2009)	1 (2009)	1 (2011)	1 (2011)	4

^a^: Information on SLPs is not provided in order to protect the confidentiality of respondents

^b^: Composite TIs are TIs that serve both FSWs and MSM/TGs

Data collection activities took place between December 2010 and May 2013, covering all three tranches of transition. Eighty-three semi-structured in-depth interviews (IDIs) and 45 focus group discussions (FGDs) were conducted across the 15 TIs. For each TI, IDIs were conducted staff from the TI, SLP, SACS and TSUs, and FGDs were conducted with KPs and PEs. This was done to gain a 360° perspective of the transition process; however, given the focus of this paper, findings presented here are restricted to those from KPs and PEs.

**Fig. 3:**
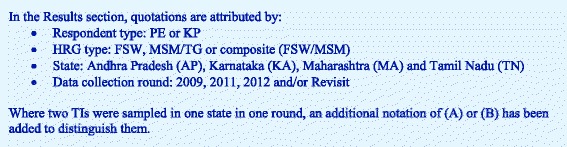
Quotation attributions

The IDIs and FGDs were recorded, transcribed and translated to English in India, and reviewed by Indian researchers for accuracy and completeness. It is worth noting that it was not always possible to disaggregate respondents from TIs that served multiple types of KPs (e.g. both FSW and MSM/TGs); where possible, the specific HRG is noted in the quotation attribution (see Fig. 3).

Qualitative data were coded and analyzed using codes based on the research plan and interview guides focusing on issues of transition preparedness, changes to services, smoothness of transition, lessons learned and relationships between stakeholders. Additional codes were developed inductively based on emergent themes in the data. Coding was conducted by two coders using Atlas.ti (Scientific Software Development GmbH) and output was reviewed by the Indian researchers who had led data collection. Inter-rater reliability checks were conducted between the coders. Analysis was conducted by TI, across each round of data collection and across the entire data set. Analysis and interpretation was iterative with each round of data collection. Ambiguities were reviewed and resolved by the whole research team collaboratively.

In addition to the interview and FGD data, documentary sources and service delivery data were also collected for all of the TIs in the sample, which were then triangulated with the qualitative data. Given the focus of this paper on the beneficiary community, these additional data sources are not drawn upon directly here, but broadly inform the analysis.

The overall evaluation was submitted for ethical review to the Johns Hopkins School of Public Health Institutional Review Board and was exempted. Within India it was reviewed and approved by the YRG Care Institutional Review Board. Informed consent was obtained from all study respondents prior to their participation. For the IDIs with program staff, written consent was obtained using consent forms written in English and the local language. After reviewing the consent form, the respondent signed the form along with the interviewer and an additional witness. For the FGDs with KPs and PEs, consent was obtained verbally due to limited literacy among respondents. The nature of the study and its risks and benefits were explained to the participants through local language interpreters. Verbal consent was sought and recorded prior to the FGD.

## Results

Three main categories of results are covered here: (1) the experience of transition, (2) changes to clinical services and (3) changes in community outreach and mobilization.

### Experience of the transition process

Three key areas emerged around the mechanics of transition itself, including (i) communications about transition, (ii) employment issues for PEs/outreach workers (ORWs), and (iii) the effects of splitting TIs on the community.

#### Communications about transition

With the exception of Tamil Nadu, communications about transition to KPs and front line staff were minimal and inadequate in the first round of transition in 2009. In some cases, respondents indicated that they only became aware of the handover when the nature of the TI activities changed.

We send them [KPs] without providing any food or beverages to them. We are feeling bad for that. That is when we came to know that [SLP] has changed to [SACS]. [PE–KA 2009]

However, during subsequent rounds, greater efforts were made to inform KPs and PEs earlier in the transition preparation period, primarily through meetings onsite at the TI.

We came to know it when we went for meeting in the office…say about two, three months. [KP (FSW)–MA 2011]

I: When did you know that this project has been shifted from Avahan to SACS and who told you about this?

R: Madam we knew about a year ago. Now we are all of the opinion that this is better now.

R: Our madam, that is ORW [NGO outreach worker], she will tell us about everything and she told that this change has taken place. [PE–AP 2012 (A)]

In Tamil Nadu, the SLP was widely credited with ensuring that PEs and KPs were informed about transition well in advance of the actual handover in all three rounds of transition.

Six months back they were telling that we are going to change to government and will be for one year or two years and if we do our profession properly, we could be hopeful enough to get some benefits as it is going to the government. And so they came to us and advised us to do our work sincerely. [PE–TN 2011]

It is important to note that across rounds and states, even when respondents were aware that the program had transitioned to government, they did not fully understand the nature of the transition and its implications. When pressed, their understanding of the technical changes to the TI and its services were vague. A notable exception was one TI in Tamil Nadu where respondents could clearly identify post-transition changes to the program.

I: How did they [the SLP] prepare you?

R: They said to us that we will get lot of benefits after taken by the government. After taken by the government, we should bring more STI tests and other things to our [Target People]. And also said we should come forward to bring more new KPs to our organization.

R: They said that blood tests are not done here. But it can be done in GH [General Hospital] after this change, they said. [KP (MSM)–TN 2011]

#### Employment issues

The transition of TIs from Avahan brought new government literacy and education requirements for front line staff that few existing PEs could meet. In fact, these new requirements made it difficult to recruit PEs from the community due to low literacy rates among KPs. The new requirements were in part due to revised reporting forms which required greater literacy. Often, outreach workers were demoted to PEs because they could not meet the new education requirements.

Another critical issue is that of PE salaries and the capping of travel allowance. PE salaries were often reduced as part of pre-transition alignment and have stagnated since. Under Avahan, PEs received an allowance commensurate with the travel they undertook to identify and meet the KPs in their catchment area. However, under government, travel costs are fixed and lower than with Avahan, so PEs must cover any extra travel from their salary, which is especially burdensome in rural districts. This change in travel allowances was viewed by front line staff as an additional reduction in their income. In general, salary and allowance changes also resulted in turnover among PEs, job dissatisfaction, and frustration that their obligations are equivalent to a full-time job without full-time pay. Fewer issues due to the salary and allowance changes were reported in Tamil Nadu, but this may be due to a negotiated agreement between the SLP and government that allowed for different PE:KP ratios for urban and rural areas (1:60 vs. 1:35, respectively) and a “group approach” whereby a whole group of PEs is responsible for a stipulated number of KPs, making monitoring more flexible. Both in Maharashtra and Tamil Nadu respondents made an effort to stress that despite the limited salaries, they continued to be committed to the work of the TI.

[I] have to spend nearly 20–50 rupees per person to come here from [district name]. For each person nearly 100 rupees have to spend to and fro. Have to spend 400 rupees for four visits. Again for roaming in local sites also we have to spend money. TA [travel allowance] are not in a proper way. Even salaries are also minimal. [PE–AP 2011]

I: So after transition… are there any people who left out due to low salary or anything like that?

R: Everybody complains for low salary but nobody wants to leave…

R: We get respect in this work… and people listen to what we speak.

R: Now we also feel proud that we are working for government.

R: One cannot survive in such a meager amount of 1500… we can get 3000 monthly for making food once in a day… but if we will think in terms of money only, then we can’t work… we feel happy to provide information to new girls… if she feel happy then we also feel happy… since we don’t want them to face problems like we did… otherwise one cannot survive with such a meager amount. [PE–MA 2012 (A)]

#### Splitting TIs

NACO generally limits the target population of each TI to 1500 KPs (though this varies by HRG type and state), while Avahan’s limits were much higher. Further, as the program evolved, HRG-specific TIs were preferred under government norms in contrast to the composite TIs (TIs targeting more than one HRG) that existed under Avahan. As a result of these standards, many TIs were split around the time of transition to meet the new KP limits and to separate HRGs; the initial number of 136 Avahan TIs resulted in over 200 transitioned TIs.

There are a limited number of TIs in this study that underwent a split; however these splits had various consequences. For example, in one composite Karnataka TI, the split was challenging because MSM/TG did not have sufficient capacity to manage their own newly-formed TI and their funds needed to be routed through the FSW TI, which caused friction. MSM/TG were left feeling resentful and unwelcome. In Maharashtra, the splitting of an FSW TI was coupled with a reorganization of the new TIs’ boundaries to better conform to the geographic realities of the area. PEs welcomed the change because it reduced their travel burden.

### Changes to clinical services

Across states, the transition to government included a planned shifting of clinical services from TI-based delivery to delivery primarily through government clinics. However, interviews revealed significant variations in how KPs experienced this change.

#### Mainstreaming and improved efficiency in clinical services

In a number of TIs across states and transition rounds, KPs described the shift to accessing clinical services within government health facilities as positive. Aspects of this shift appreciated by many KPs included a sense of “mainstreaming,” associated with a greater confidence attending facilities used by the general public. Specific benefits identified by KPs included greater availability of clinicians and medicines, improved linkages to other services, and more streamlined and efficient care (e.g., a single blood test for HIV and syphilis).

R: Since the clinics are being conducted at the government hospitals, our community women have all the advantages. R: All will be there, won’t they, Sir? ICTC [Integrated Counseling and Testing Center] Counselor, lab technician and the doctor, all will be available at a single place. [PE–KA 2011 (B)]

Everything is there. It is better than that, because first they were giving us only STD-TB treatment. We were getting treatment for sexual diseases only from our service organization. After the government took over, we are getting what the ordinary public is getting. Actually we are getting more than what they get. So after the government has taken, the KP [s] are getting more of everything. [PE–TN 2009]

Related to this, numerous KPs across TIs also described receiving improved interpersonal care at government facilities; they linked this to factors including a stronger sense of entitlement among KPs, a greater sensitization of providers and other staff to the needs of HRGs, and an increased sense of accountability within government services. These improvements were sometimes attributed to the transition itself, and sometimes to other initiatives being implemented by the government or local NGOs/CBOs, including KP-led advocacy.

R: It’s ok…it’s better now than before it was. Lawyers, police, all of them talk to us with respect. They say…‘madam is coming’…it’s good.

R: Earlier, they looked down upon us…‘oh…these people do such disgusting jobs? How can we touch them?’ Such was their feelings about us. They ill-treated us. Now it’s different. They welcome us and offer us a seat. If we take somebody else along with us, they too are treated with same respect. We are getting better treatment now. [KP (FSW)–KA 2009 Revisit]

#### Concerns about “blending in” and disclosure

While most KPs described more sensitive treatment from government health providers following the transition, some KPs expressed mixed feelings about this, including a fear of disclosure of STI or HIV status. While TGs expressed particular concern about not being able to “blend in” at public health facilities, some FSWs also expressed fear regarding confidentiality.

In [SLP] when they detect us positive they used to maintain the secret and no one else would know about that. But now when we go there in government hospital, it is getting spread. People see us and think that we are these sorts of people. Even those people who are all friendly with us are moving away from us. We are getting a bad name in the society…They come to know that we are transgenders…it becomes a problem. When they see us they will keep a distance of 10–15 feet away from us. Even the sisters who come to take blood would give such a cheap look. [KP (MSM/TG)–TN 2011 Revisit]

R: Earlier a van used to come for ICTC tests then very slowly we use to go to that van by pretending as if we are going somewhere from that tree side or something else. We use to jump in to the van very fast…We use to go for that blood test without notice to any other person. But it is not like that madam.

I: Do you have to go to center?

R: Yes madam, we have to cross the ten members crowd and approach that shop. Then people will notice us and might think that we are bad people. That is the fear that we have in our mind. [KP (FSW/MSM)–AP 2011]

#### Difficulties accessing government clinical facilities

KPs and PEs from several TIs complained that the transition led to more difficulty in accessing health services, due to increased distance to government facilities, inadequate staffing/stock at these facilities, language barriers, inconvenient hours of operation, and longer wait times or more rigid rules regarding appointments.

Earlier there was care and medication…now we do not get anything. They just give the prescription to buy it from the market…how can they provide it, when they do not have it in their stock? [KP (FSW)–MA 2011 Revisit]

R: If they give the treatment the same day it will be all right, but they will ask us to come the next day. We are leaving our work and going there…And so we will lose one more day’s work. But it was not like that when it was in [SLP]. When we go there they will give all the medicines, treatment, and advice that we require. [KP (MSM/TG)–TN 2011 Revisit]

R: Madam we belong to Bangladesh…we all speak Bengali…but nobody pays attention over there [at government clinic] since they pay attention to the Marathi speaking people…we don’t know the procedure of filling forms also…we can’t speak Hindi or Marathi fluently, we can speak in our language only…now doctors don’t pay much attention to us over there. [PE–MA 2012 (A)]

#### Loss of general health services

In numerous TIs, including those where KPs felt that clinical services had improved, many respondents criticized a loss of general health services offered through the TI, including for their children.

Previously there were many services and we did not have any problems. But after the transition to the government, the services have come down. The services have been reduced…if their children were sick they were free to take them for treatment. But now after we joined [SACS], we did not receive anything. Just they allow us to take test and to test HIV. They are giving importance only for this. [PE–TN 2011]

Numerous KPs described a perceived narrowing of focus after the transition, with emphasis only on ICTC and condom distribution and reduced support for other sexual health and STI services, including a shift away from treating sexual partners.

R: When [SLP] was there, partner treatment concept was there. For every three months partners were brought for treatment. Now it is not like that. Partners are asking about that…Our opinion is also that it is good if the concept continues. [KP (MSM/TG)–AP 2009]

#### Changes in government commodities

Across TIs there was considerable criticism regarding government commodities, particularly condoms and lubricant. Many KPs felt that the government condoms were worse than those they had received earlier from TIs for reasons including bad smell and taste, lack of lubrication, inappropriateness for anal sex, and indiscreet packaging. Related to the challenges discussed above, some KPs also complained that commodities such as anti-itch ointment, pregnancy tests, and painkillers were no longer available.

R: No one from the community use condoms as they used to do earlier. Sometimes there is a bad smell. Sometimes the expiry date is over…

R: This one breaks as soon as we put it on the cock. If we use 2 or 3, then it is ok.

I: Was this problem not there during earlier time?

R: No, earlier they were of good quality. No smell was there. It was enough to wear just one. But it is not so now. Even if 3 are used at a time they break open. [KP (MSM/TG)–KA 2012]

R: The old one was with fragrance and black in color, in packing they looked like shampoo packs, it was not easy to guess them. The new ones you cannot keep them anywhere. The old ones were good in this sense.

R: They are not good in this sense that you have to take care with them, you cannot leave them lying anywhere as even the kids can read the name, so you have to hide them. [KP (FSW)–MA 2011]

While most comments regarding government commodities were negative, some KPs expressed appreciation for government condoms as well as newly available commodities, particularly female condoms.

### Targets for clinical services

In a number of TIs, respondents described an increase in frequency of referral and service contacts following the transition. Some KPs considered this a positive change, describing greater engagement by PEs, including more intense follow-through to ensure that testing referrals were completed.

Earlier, PEs met us at only at some sites and not in all places. When we community people meet the PEs, maybe at the site they never used to talk, they used to work by themselves, and be on their way when done. Now it is not that way at all. If PE meets anyone of our community they talk to us nicely and ask after our health. They take us along for ICTC…once in every six months. They come along with us; after ICTC if we have any problems, counselor asks us to come after half an hour or one hour. They give the report to us and our peers give us complete information on it and send us back home. [KP (MSM)–KA 2009 Revisit]

However, some PEs perceived this increased contact as pressure to accept HIV testing, partly driven by government targets for clinical service delivery. The combination of testing targets with a loss of general health services was deemed to make it doubly difficult to engage KPs.

R: When there was [NGO] previously, we were not forcefully bringing women to the clinics. We had no targets. Now targets have been given. Women should get ICTC and clinical tests done is what they insist now…

R: Now, every six months ICTC is being done. If we go again and ask, they [KPs] feel it is too early for them and say just recently it was done. They are worried if we visit them every six months. They feel that we are insisting ICTC very often. [PE–KA 2011 (A)]

### Variation over time

Some of the negative perceptions of clinical services expressed by KPs lessened between the initial and revisit rounds. For instance, respondents from a TI who had originally been uncomfortable using government health services reported broad satisfaction with these services at the time of revisit.

When in [SLP], clinic was here only…With a view to mingle up with the society, we are being referred to the government hospital. Some felt difficulty with this. [Before the transition], only one doctor was coming to us…Any confidential related issues were discussed here and they didn’t feel shy for going for check-ups [at the TI]…Once when they were referred to the government hospital, some complaints have been reported to go in for check up with the big group of people. But slowly this was also habituated and the disparity also lessened. The services there are also rendered very well. [PE–AP 2009 Revisit]

While respondents from a number of TIs continued to describe similar problems during the initial and revisit interviews, very few issues became worse.

### Changes to community outreach and mobilization

Much of the discussion around community mobilization activities came from institutional actors, including the SLP, government and the TI managers. Community members, both KPs and front-line staff, often did not differentiate between outreach activities and mobilization efforts as they saw these all as part of the TIs efforts to benefit the community as a whole. In general, state-level respondents acknowledged that SACS did not see a clear role for themselves in establishing CBOs or in capacity building for community members and did not feel equipped to support community mobilization and outreach adequately. Accordingly, responsibility for mobilizing the community fell on the SLP and TI managers as part of the post-transition support agreements that were made for each transition round. This section elucidates the changes perceived by KPs and PEs in community outreach and mobilization.

#### Community events

Prior to the transition, socially-valued community events were a time to come together and celebrate. The events involved dancing, storytelling and entertainment, and the PEs were able to communicate health messages through a variety of creative outlets. However, community events have decreased since the transition, likely due to the decreased funding under SACS.

They would provide us information on MSM and conduct theatre and MSM programs. They would do it in a proper way. Now those programs are not conducted. There would be lot of people coming to the MSM office for the theatre program. Lot of people would gather. Everybody would be happy. Now there are no programs conducted. [KP (MSM/FSW)–KA 2009]

Furthermore, there was a strong and frequently expressed feeling among KPs and PEs that TI emphasis has shifted to clinical services only, and that activities that brought the community together had been abandoned. KPs expressed dissatisfaction at the elimination of ancillary services, such as hair salons, and celebrations around festivals, holidays, or special events in each other’s lives, such as baby showers for transgender KPs. These had provided recognition missing from KPs’ daily lives and a safe space in which to celebrate rituals.

R: Sir, previously they used to call for…Srimanthas [a festive rite done in particular months of pregnancy] used to be conducted monthly. We all used to meet and discuss about any problems we have.

I: Now what is happening?

R: Now nothing is there of that sort sir. [KP (MSM/TG)–AP 2009 Revisit]

We used to come while [SLP] was there. At the time there was more focus on bringing us together rather than on health issues. We had meetings, activities and all that. We had dance programs every three months and DIC [Drop in center] meetings twice in a month and whatever the occasion, meetings or anything, we were provided with good, satisfying meal and they were giving trainings to the entire community. We were taken to places like [district] and others and given training. But it is not that way now. [KP (MSM)–KA 2009 Revisit].

#### Incentives for engaging in TI activities

Under Avahan, several efforts were made to engage KPs, including providing tangible incentives for participation. Across all states the outreach budget decreased under SACS leadership, resulting in the elimination of small incentives, such as refreshments or small gifts. Providing incentives to the KPs encouraged them to visit the TI or engage with the PEs; removing them has dampened interest for KP participation.

Earlier so many KPs used to come here. They were provided with food etc. Now they want rent also, but we don’t want to give right now. Now food is also not provided. Now see, when we were called in the morning, we asked KPs also to join. They didn't have their food and came here thinking that ‘when we will go there, we will get food also’, so they come, but they don’t get. Therefore, they think that there is no fun going there. ‘Shouldn’t we continue our activities rather going there?’ They speak like this. [PE–MA 2011 Revisit]

#### Community responses to structural violence

Across all states and all rounds of transition, KPs and PEs highlighted police brutality and police raids as examples of structural violence faced in their everyday lives. In many contexts, the TI provided crisis management services and police advocacy to resolve disputes, and these services were maintained after the transition. Crisis management support was important to sex workers, who were encouraged to call for support during a police incident or other violent encounter, including domestic abuse. These services were considered particularly important for those who did not speak the language, such as migrant sex workers. These activities continued post-transition and both the PEs and KPs were generally satisfied with the collaborative support that they received. Crisis management efforts were supported by the government, but generally implemented by the SLP or a CBO after the transition.

In case of any emergency or crisis we should assure them [KPs] the support. If there are any major issues, we shall deal them within 24 hours. In case of minor crisis, we will try to solve them on the same day…Through all these activities we have built up a good rapport outside. The organization [NGO] has become strong. We were trained how to go and speak to the women in times of need. We never knew how to talk to support the leaders and compromise them etc. before the training. [PE–KA 2011 (A)]

Earlier it was not so that whenever we came across someone hitting the other, someone abusing, someone insulting, someone else is involved in quarrel, we were not able to contact each other or see each other, but now we people are able to cooperate each other…Now we are not afraid of police even, we can argue with them even to some extent. [PE–MA 2011 Revisit]

Discussions from Andhra Pradesh, Karnataka and Tamil Nadu suggest that harassment by police has markedly diminished since the transition. Improvements since the transition may be credited to the SLP or CBO maintaining crisis support services as part of the post-transition plan (with support from SACS in some states), including sensitization activities with the police.

No madam. They [SLP] are doing everything for us whenever we demanded them. They have introduced [crisis management] scheme for us which will help us to solve our problem within 24 hours. But, nowadays, we get the solution for our problems within 12 hours in very respectable and responsible manner. Even though our programs are undertaken by the government, [SLP] still continues its support for us. They never gave up in any circumstances. They were doing everything for us and still they are continuing it and they will also. [KP (MSM)–TN 2011]

In Maharashtra, respondents indicated that police brutality and violence from the community at large has continued post-transition, with PEs doing their best to intervene on the behalf of KPs.

I cannot allow this [harassment by police] with my KPs. Sometimes if I find this type of problem, firstly I go and stand in front. ‘If you have to fight, to speak, speak to me what you want to speak’. One day the leader from there, come and ask me how I show so much power, I said that I have collected. If they tortured my KPs, I will not leave them, because they are earning for their stomach and eating. [PE–MA 2011]

## Discussion

The findings from this study highlight three areas of the beneficiary community experience of transition. Communication with KPs and PEs was inadequate in the first tranche of transition but improved over time, whereas employment issues, characterized by stagnating salaries, loss of travel funds and changes to education requirements, persisted as a challenge throughout the transition. Further, the splitting of TIs as part of alignment pre-transition uncovered an underlying imbalance in the capacity of different HRGs that had not been observed before, with MSM/TG TIs generally weaker than FSW TIs. In terms of clinical changes, KPs were directed to government clinics following transition, which highlighted contrasting experiences depending on HRGs. Many KPs welcomed the mainstreaming that came with this shift as they sensed that government services were more accepting than previously. However, others—especially TGs—worried about being able to blend in with the general population at these centers. Other practical issues arising from the move to government clinics included increased distance to facilities, inconvenient hours, and loss of other health services valued by KPs. Finally, the changes that took place for community outreach and mobilization, including loss of social events, refreshments and other incentives, were viewed negatively by the beneficiary communities across the study TIs. These changes impacted the KPs’ perceptions of the program, their interest in attending activities, and underlined the shift in focus of the TIs’ mission.

A key theme emerging from the findings above is that KPs perceive the TIs as shifting from being about the KPs and their needs to being about HIV/AIDS only. Respondents described a sense that the TIs’ previous holistic approach, including general health services, empowerment activities and social engagement, had been lost in favor of an emphasis on HIV/AIDS-related clinical services, particularly HIV testing and condom distribution. This theme resonated across states, TIs, and transition rounds. Even in settings where most KPs praised an overall improvement in clinical services, they expressed regret at the discontinuation of or reduced support for community events and non-clinical services that had been appreciated in the past–such as hair salon services at drop-in centers and celebrations culturally coded as feminine for TGs (e.g., baby showers). The cessation of benefits like refreshments at TIs also contributed to a sense that building social cohesion or showing respect to KPs was de-prioritized in favor of a target-driven monitoring of clinical service uptake. These incentives had functioned as a form of politeness or respect not usually extended to marginalized populations in mainstream venues. Similarly, the loss of support for travel costs or compensation for lost work time added to the perceived costs incurred by KPs for participating in TI programs post-transition.

The available data do not allow us to assess whether this shift in focus and loss of ancillary benefits may lead to a reduction in service uptake. Nevertheless, the fact that criticism regarding these changes was expressed by respondents across very different settings suggests a profound difference between the approach of Avahan-based HIV prevention activities and those operated by the government. Avahan-affiliated TIs aimed to deliver HIV/AIDS prevention services through a lens of social mobilization that united KPs and created a safe space for these marginalized groups to congregate. However, SACS and government-run HIV/AIDS facilities were established for the primary purpose of delivering HIV/AIDS services and commodities. The difference between these two approaches may mean that, no matter how smooth a transition has been from the institutional perspective and regardless of the effect on service uptake, there will be an inevitable sense of disruption or loss for KPs. As such, our findings are consistent with other studies in India that suggest that more holistic approaches that incorporate community empowerment and mobilization may have a positive impact on HIV prevention, particularly for groups facing structural vulnerability such as FSWs [10–12].

The loss of a “one-stop shop” model of holistic services may have, in fact, created additional opportunity costs for KPs who now have to assemble all the activities they value from different venues. There are numerous government efforts in India to provide relevant services, such as general health services, national identity cards, affordable housing, nutrition support, and education, but they may not be well-coordinated. In some states, there are also larger efforts to address the needs of specific KPs, such as the Transgender Welfare Board in Tamil Nadu.

In fact, NACO had recognized the value of broader support for HIV prevention and engaging communities in NACP-III. NACP-III encourages linkages with community development and social entitlement programs, such as vocational training and micro-credit groups, as well as with the National Rural Health Mission (NRHM) for access to reproductive and other health services [1]. These efforts, while commendable, have been difficult to implement, and a number of challenges have been identified, including difficulty in the functional integration of services with the NRHM and link worker schemes [13]. Similarly, the NACO target for 50 % of TIs to be led by CBOs after transition was not met due to lack of capacity among communities and CBOs [14]. The findings from this study further underscore the gap between national-level policy making taking place within NACO and its partners and how those policies are implemented at the state and district levels. Targets and higher-level policy documents can help establish an expectation, but there needs to be recognition of the time it takes to develop sufficient capacity at the community-level as well as the potential need for state-level government actors to develop their own capacity to act as a resource for this process.

There are several limitations to this study. First, although these case studies were selected purposively to capture as much variation as possible, the sample resulted in generally only one TI per state for each tranche of transition. However, given the consistency of responses across settings, we are confident that the results reflect experiences common across the Avahan program. Second, there were challenges for maintaining consistency among respondents given the turnover taking place at the community level, especially for TIs that were revisited long after transition. Given their dual role, it was at times difficult to distinguish whether PEs were responding in their capacity as a KP or as a staff member. Lastly, we did not collect service delivery data before or after transition. This has limited our ability to understand how service provision functioned pre-and post-transition or how they might have changed. We have tried to address this by triangulating the qualitative data presented here and the programmatic data that had been collected alongside.

## Conclusions

While we are unable to draw conclusions regarding the Avahan model elements that are truly critical to successful HIV/AIDS prevention, as opposed to those that may be desirable from the perspective of KPs but are not essential, future donor transitions can draw some concrete lessons from this study, whether in the area of HIV or not. Even when the specific programmatic changes cannot be predicted, early planning is essential. The involvement of beneficiary communities in the planning process can mitigate feelings of loss and potentially facilitate a constructive dialogue between all stakeholders. While transitioning a program from one set of funders and managers to another can be a monumental task, the inputs and engagement of those served by the program remain crucial to its long-term success.

## Abbreviations

AP: Andhra Pradesh
BMGF: The Bill and Melinda Gates Foundation
CBO: Community-based organization
FGD: Focus group discussion
FSW: Female sex workers
HRG: High-risk group
ICTC: Integrated counseling and testing center
IDI: In-depth interview
KA: Karnataka
KPs: Key populations
MA: Maharashtra
MSM: Men who have sex with men
ORW: Outreach worker
NACO: National AIDS Control Organisation
NACP: National AIDS Control Program
NGO: Non-governmental organization
NRHM: National Rural Health Mission
NTSU: National technical support unit
PE: Peer educator
SACS: State AIDS Control Society
SLP: State lead partner
STI: Sexually transmitted infection
TG: Transgendered individual
TI: Targeted intervention
TN: Tamil Nadu
TSU: Technical support unit

## Footnotes

1 The interventions located in the Northeast of India transitioned on a much later schedule and were not included in the overall evaluation.
